# Under the armor: X-ray computed tomographic reconstruction of the internal skeleton of *Coahomasuchus chathamensis* (Archosauria: Aetosauria) from the Upper Triassic of North Carolina, USA, and a phylogenetic analysis of Aetosauria

**DOI:** 10.7717/peerj.4368

**Published:** 2018-02-13

**Authors:** Devin K. Hoffman, Andrew B. Heckert, Lindsay E. Zanno

**Affiliations:** 1 Department of Geosciences, Virginia Polytechnic Institute and State University (Virginia Tech), Blacksburg, VA, United States of America; 2 Department of Geological and Environmental Sciences, Appalachian State University, Boone, NC, United States of America; 3 Department of Biological Sciences, North Carolina State University, Raleigh, NC, United States of America; 4 Division of Paleontology, North Carolina Museum of Natural Sciences, Raleigh, NC, United States of America

**Keywords:** Aetosauria, Aetosaurs, Specimen-based phylogeny, CT reconstruction, Late Triassic, North Carolina, Carnian

## Abstract

Aetosauria is a clade of heavily armored, quadrupedal omnivorous to herbivorous archosaurs known from the Late Triassic across what was the supercontinent of Pangea. Their abundance in many deposits relative to the paucity of other Triassic herbivores indicates that they were key components of Late Triassic ecosystems. However, their evolutionary relationships remain contentious due, in large part, to their extensive dermal armor, which often obstructs observation of internal skeletal anatomy and limits access to potentially informative characters. In an attempt to address this problem we reanalyzed the holotype of a recently described species of *Coahomasuchus*, * C. chathamensis*, from the Sanford sub-basin of North Carolina using computed tomography (CT). CT scans of the holotype specimen clarify preservation of the skeleton, revealing several articulated vertebrae and ribs, an isolated vertebra, left ulna, left scapula, and the right humerus, though none of the material resulted in updated phylogenetic scorings. Reexamination of aetosaur materials from the holotype locality also indicates that several isolated osteoderms and elements of the appendicular skeleton are newly referable. Based on these results, we update the *Coahomasuchus chathamensis* hypodigm and conduct a revised phylogenetic analysis with improved character scorings for *Coahomasuchus* and several other aetosaurs. Our study recovers *Coahomasuchus* in a polytomy with *Aetosaurus* and the Typothoracinae, in contrast with a recent analysis that recovered *Coahomasuchus* as a wild-card taxon.

## Introduction

Aetosaurs comprise a clade of heavily armored, quadrupedal herbivorous to faunivorous pseudosuchian-line archosaurs (Parker, 2016a; Heckert, Fraser & Schneider, 2017) known from Upper Triassic deposits across Pangea. To date, fossil specimens have been recovered from every modern continent except Antarctica and Australia (Heckert & Lucas, 2000; Desojo et al., 2013; Schoch & Desojo, 2016; Heckert, Fraser & Schneider, 2017). As one of the few lineages of non-dinosauromorph archosauromorphs to have evolved herbivory during the Triassic (Desojo et al., 2013), aetosaurs represented a key component of Late Triassic ecosystems.

**Figure 1:**
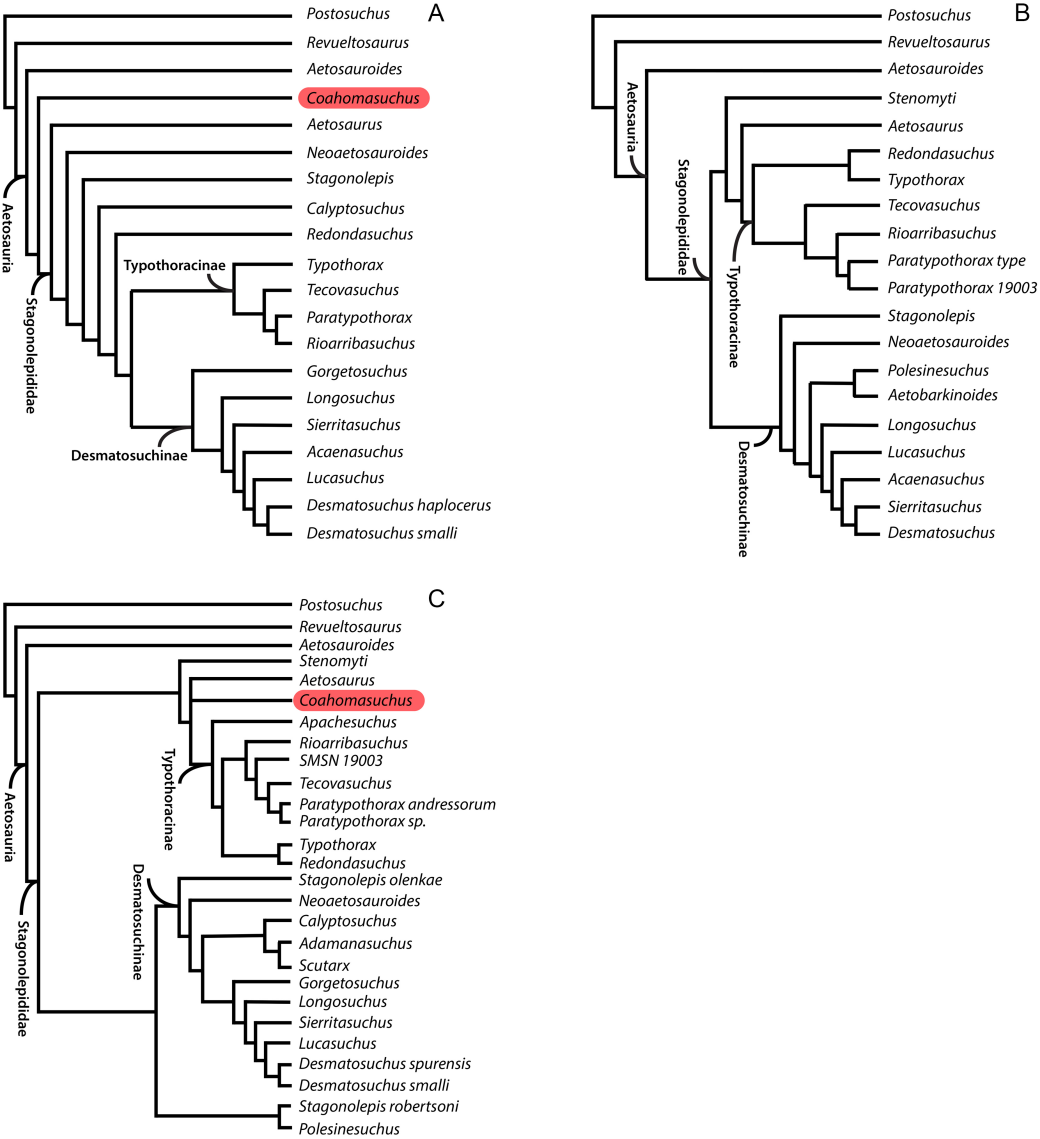
Comparison of recent phylogenetic analyses of Aetosauria. Comparison of the phylogenetic position of *Coahomasuchus* in three recent aetosaur phylogenetic analyses. (A) *Coahomasuchus* found outside of Stagonolepididae (Heckert et al., 2015); (B) *Coahomasuchus* found to be a wild card taxon and pruned from final analysis (Schoch & Desojo, 2016); (C) *Coahomasuchus* recovered in a polytomy with *Aetosaurus* and Typothoracinae (Parker, 2016a).

Understanding aetosaur phylogenetic relationships and skeletal anatomy are important for building the bigger picture of archosaur diversification during the Triassic. However, aetosaur phylogenetic analyses are complicated by the abundance of osteoderms in individual skeletons, often found dissociated from each other and the rest of the skeleton and that suffer from character homoplasy, which have been used as the basis of character scorings for this group (Heckert & Lucas, 1999; Heckert & Lucas, 2000; Harris, Gower & Wilkinson, 2003; Parker, 2007; Parker, Stocker & Irmis, 2008; Desojo, Ezcurra & Kischlat, 2012; Desojo et al., 2013; Parker, 2016a). Because of this armor, the internal skeletal anatomy of many species is poorly understood, as the best preserved aetosaur specimens are articulated (e.g., NMMNH P-18496, *Coahomasuchus kahleorum*, Heckert & Lucas, 1999; NMMNH P-56299, *Typothorax coccinarum*, Heckert et al., 2010), and thus the osteoderms obscure much of the appendicular and axial skeleton including, sometimes, features of the osteoderms themselves. Indeed, even in cases of spectacular preservation of multiple, articulated individuals, such as the SMNH *Aetosaurus* block, the presence of so much armor obscures details of the appendicular skeleton (e.g., Schoch, 2007). Additionally, some aetosaur taxa (e.g., *Apachesuchus heckerti, Gorgetosuchus pekinensis, Redondasuchus rineharti, Rioarribasuchus chamaensis*) are known exclusively from osteoderms (Desojo et al., 2013; Heckert et al., 2015; Parker, 2016a), and can only be partially scored into phylogenetic analyses. Even those aetosaurs known from relatively complete materials, such as *Coahomasuchus* suffer from instability in recent phylogenetic analyses, which may stem from conflicting phylogenetic signals between different morphological character partitions (Parker, 2016a). The three most recent phylogenetic hypotheses to include *Coahomasuchus* (Heckert et al., 2015; Schoch & Desojo, 2016; Parker, 2016a) each posit a different phylogenetic position for the genus relative to other aetosaurs (Fig. 1).

**Figure 2:**
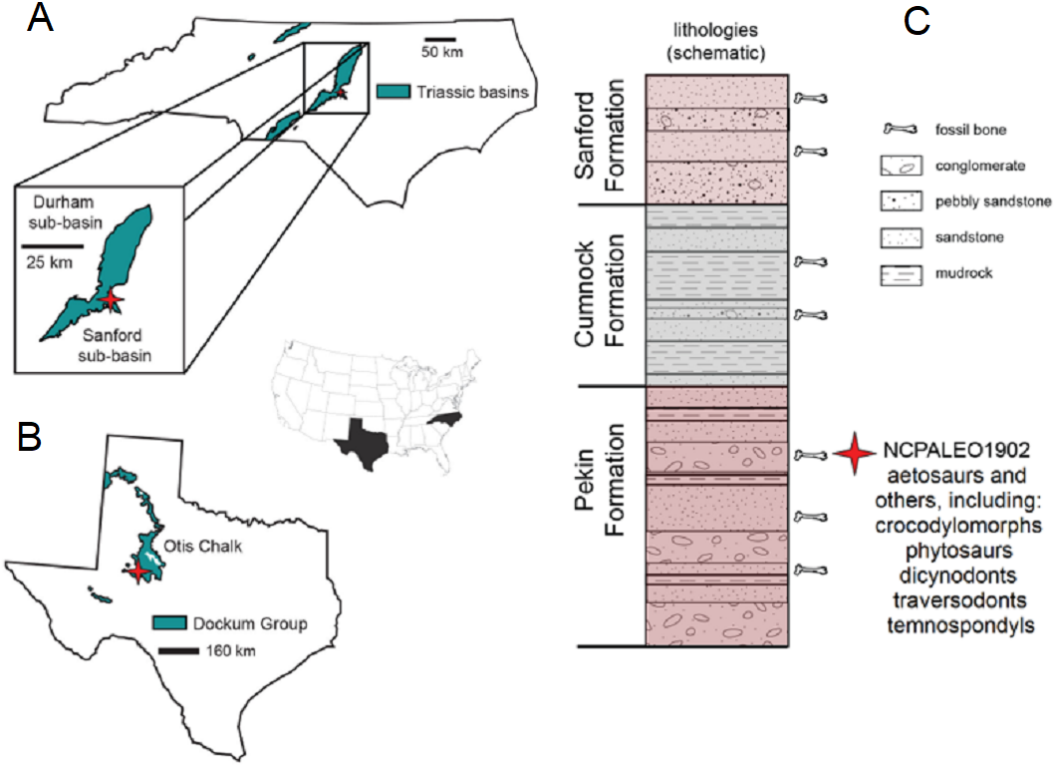
Locality information and stratigraphic position of NCPALEO1902. (A) Generalized map of North Carolina’s Triassic basins showing the location of the *Coahomasuchus chathamensis* holotype specimen within the Deep River Basin, modified from Heckert, Fraser & Schneider (2017, fig. 1.1). (B) Generalized map of the Upper Triassic strata of Texas showing the locality of original *Coahomasuchus kahleorum* specimen (NMMNH P-18496), based on Heckert & Lucas (1999, fig. 2); (C) simplified stratigraphic section of the Sanford sub-basin showing the approximate stratigraphic position of NCPALEO1902 and the type locality of *Coahomasuchus chathamensis*, from Heckert, Fraser & Schneider (2017, fig. 1.3).

**Figure 3:**
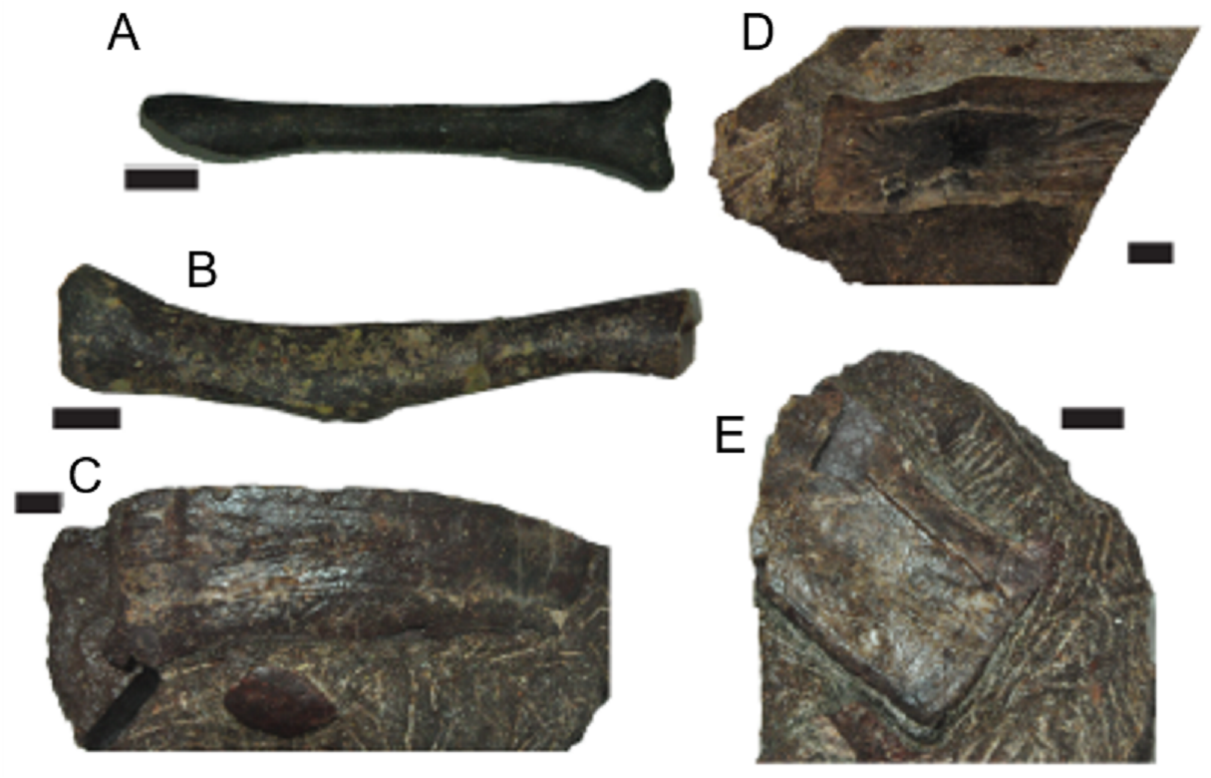
Select additional referred material of *C. chathamensis*. (A) NCSM 19765 (radius); (B) NCSM 16441 (fibula); (C) NCSM 20827 (paramedian osteoderm); (D) NCSM 21602 (paramedian osteoderm); (E) NCSM 26204 (paramedian osteoderm).

*Coahomasuchus* is currently represented by two species, *C. kahleorum* (Heckert & Lucas, 1999) and *C. chathamensis* (Heckert, Fraser & Schneider, 2017). *Coahomasuchus kahleorum* is known from one published specimen, the holotype skeleton (NMMNH P-18496), found near Coahoma, Texas in the Upper Triassic Carnian (Otischalkian) Colorado City Member of the Dockum Formation of the Chinle Group (Heckert & Lucas, 1999). The type specimen is a nearly complete, articulated skeleton approximately 71 cm long as preserved, with complete osteoderm sets from the cervical series to the middle of the tail, the braincase, parts of each limb and their respective girdles, appendicular osteoderms, and much of the vertebral column (Heckert & Lucas, 1999; Desojo & Heckert, 2004; Parker, 2016a). Assuming it is a skeletally mature individual, *C. kahleorum* is relatively small-bodied, and can be distinguished from co-occurring aetosaurs by its parallel, sub-radial ornamentation (Heckert & Lucas, 1999). In Heckert & Lucas’s (1999) original analysis, *C. kahleorum* was found to be a relatively plesiomorphic aetosaur, and seen as filling the apparent stratigraphic gap or “ghost lineage” between late appearing, but relatively plesiomorphic aetosaurs like *Aetosaurus* and early forms with derived characteristics such as *Desmatosuchus* and *Longosuchus*. There is also a second, undescribed specimen, located at the Texas Memorial Museum that Parker (2016a) used to score some of the characters scored for *Coahomasuchus* in his recent phylogenetic analysis. The specimen remains under study by Parker and was not examined by us. A largely articulated presacral skeleton of *Coahomasuchus* (NCSM 23618) was recently described as the holotype of a second species, *Coahomasuchus chathamensis*, from the Upper Triassic of North Carolina (Fig. 2A). In addition to the holotype, there are a variety of specimens from the same locality that are referable to *Coahomasuchus chathamensis* (Table 1; Fig. 3). These new materials allowed us to address the lability of *Coahomasuchus* in recent phylogenetic analyses by increasing the proportion of character scorings for the genus through direct observation of *C*. *chathamensis*. All fossil specimens of *C. chathamensis*, including the holotype specimen and referred materials from the type locality, are housed in the vertebrate paleontology collections at NCSM (Heckert, Fraser & Schneider, 2017). Much of the interior skeleton of NCSM 23618 is obscured by ventral, paramedian, and appendicular osteoderms and Heckert, Fraser & Schneider (2017) speculated that additional vertebrae and elements of both forelimbs might be preserved in the holotype specimen. Here we use X-ray computed tomography (CT) to identify elements of the holotype specimen not observable via visual inspection. We also update the list of specimens referable to *Coahomasuchus chathamensis* and modify the referred list of specimens provided by Heckert, Fraser & Schneider (2017).

**Table 1:** Additional *C. chathamensis* specimens in NCSM collections. List of NCSM specimens definitively referable to *C. chathamensis* that are not noted in Heckert, Fraser & Schneider (2017). An equal number of NCSM specimens are of comparable size and referable to Stagonolepididae; all share similarities with *C. chathamensis* yet are not diagnostic of the genus and may later be assigned to *C. chathamensis* or another aetosaur.

NCSM number	Element
16368	Six associated osteoderms (only largest paramedian definitively *Coahomasuchus*)
16434	Partial left paramedian osteoderm
16435	Paramedian osteoderm
16436	Block with six osteoderms (one left paramedian)
16441	Fibula (incomplete)
16445-3	Left paramedian osteoderm
16472	Dorsal paramedian osteoderm
18709	Right paramedian osteoderm
18819	Lateral osteoderm
19302	Right paramedian osteoderm
19303	Posterior dorsal paramedian
19633	Partial lateral osteoderm
19635	Right paramedian osteoderm
19765	Radius (incomplete)
20406	Right paramedian osteoderm
20797	Right paramedian osteoderm
20799	Ventral osteoderm
20827	Partial right paramedian osteoderm
20908	Right paramedian osteoderm
21062	Paramedian osteoderm
21071	Left lateral osteoderm
21137	Left paramedian osteoderm
21175	Paramedian osteoderm
21180	Left paramedian osteoderm
21274	Left caudal lateral osteoderm and impression
21569	Right(?) lateral osteoderm
21602	Right paramedian osteoderm
21604	Left paramedian osteoderm
24808	Ventral(?) osteoderm
26014	Ventral osteoderm
26023	Partial osteoderm
26203	Partial lateral osteoderm
26204	Paramedian osteoderm

### Geologic setting

All known specimens of *C. chathamensis* come from a single locality (NCPALEO1902) in Chatham County, North Carolina (Fig. 2A). More detailed locality information is on file at the NCSM. This locality is in the Sanford sub-basin, part of the larger Newark Supergroup (Heckert, Fraser & Schneider, 2017). All of the Triassic sedimentary rocks in North Carolina were referred to the Chatham Group of the Newark Supergroup by Olsen (1997) and Weems & Olsen (1997). This assignment was based on the apparently synchronous deposition of the units in rift basins during the breakup of Pangea along nearly the entire eastern margin of North America (Weems & Olsen, 1997). The Sanford sub-basin represents a half-graben bounded by the Jonesboro fault system (normal faults) on the eastern margin (Olsen et al., 1991). This region contains three formations originally described by Campbell & Kimball (1923) as, in ascending order, the Pekin, Cumnock, and Sanford formations, all of which yield fossils (Fig. 2B). The upper and lower formations (Pekin and Sanford) are largely “red-bed,” sandstone-dominated units surrounding the Cumnock Formation of mostly gray claystone with occasional coal seams (Olsen et al., 1991; Heckert, Fraser & Schneider, 2017). Other fossils from this same locality include cynodonts (Liu & Sues, 2010), dicynodonts (Green et al., 2005; Green, 2012), the crocodylomorph *Carnufex carolinensis* (Zanno et al., 2015; Drymala & Zanno, 2016), aetosaurs, including *Gorgetosuchus* (Heckert et al., 2015), and numerous unpublished specimens.

All of the *C. chathamensis* material, and, indeed, essentially all of the published vertebrate fossils, were recovered from the uppermost portion of the Pekin Formation (Heckert, Fraser & Schneider, 2017). These fossils came primarily from a fine-grained red siltstone, and some osteoderms from a coarser-grained greywacke (Heckert, Fraser & Schneider, 2017), with many other fossils found in conglomerates and sandstones. Many of the specimens of *Coahomasuchus* described here, including the holotype of *C. chathamensis*, were found associated in monotypic assemblages of bones in blocks of unquarried material. The coarser lithologic units have been interpreted as fluvial channels or as alluvial fan deposits (Olsen et al., 1991; Heckert, Fraser & Schneider, 2017). Determining the exact stratigraphic position of these specimens in the Pekin Formation is not possible because they were collected from disturbed blocks of sediment; however, they can be assigned to the upper half, but not the uppermost portion, of the formation (Heckert, Fraser & Schneider, 2017).

As the stratigraphically lowest unit in the Newark Supergroup locally, the Pekin has long attracted interest. Palynostratigraphy has historically positioned the Pekin Formation in the Carnian stage (e.g., Cornet, 1993; Litwin & Ash, 1993), further verified by the vertebrate stratigraphic correlations made by Huber, Lucas & Hunt (1993) and Lucas & Huber (2003). More recently, much of the Newark Supergroup thought to be Carnian in age has been reassigned to the Norian stage on the basis of the “long Norian,” with a Carnian–Norian boundary of ca. 228 Ma (Muttoni et al., 2004; Furin et al., 2006). Although the “long Norian” has been questioned (e.g., Lucas et al., 2012), the most recent age estimate for the Pekin Formation comes from Whiteside et al. (2011), whose paleomagnetostratigraphic correlations with other Newark Supergroup units suggested an age of 231 Ma for the Pekin Formation. This age fits with both the “long Norian” (Muttoni et al., 2004; Furin et al., 2006) and Lucas’s (2010) Triassic timescales for the Carnian and makes *C. chathamensis* one of the oldest known aetosaurs as, to date, there are no pre-Carnian aetosaurs known (Heckert & Lucas, 2000; Desojo et al., 2013).

## Materials & Methods

### Specimen imaging

All fossil preparation was performed at NCSM following the procedure described by Heckert, Fraser & Schneider (2017). The holotype specimen NCSM 23618 was scanned twice under different parameters: a coarser resolution scan was conducted at Siemens Medical Training Facility in Cary, North Carolina; however, this scan resulted in poor quality data. A second, higher resolution scan (0.6 mm) was performed at North Carolina State University College of Veterinary Medicine Diagnostic Facility. The resulting DICOM data was processed and segmented using Avizo version 9.0.0 in the Paleontology Research Lab at the North Carolina Museum of Natural Sciences, Raleigh, North Carolina.

### Phylogenetic analysis

Schoch & Desojo (2016) used a character matrix based on the dataset of Parker (2007), as updated and modified by other authors, most recently Roberto-Da-Silva et al. (2014), with the addition of seven new cranial characters to explore the evolutionary relationships of *Paratypothorax*. They found *Coahomasuchus* to be labile within Aetosauria, but their analysis lacked several recent character changes published by Heckert et al. (2015) of *Coahomasuchus* and other aetosaur taxa, including *Lucasuchus, Longosuchus*, and the recently described *Gorgetosuchus*, although Heckert et al.’s (2015) analysis did not include *Stenomyti huangae*. The recently described species, *C*. *chathamensis* (Heckert, Fraser & Schneider, 2017) allows for several new characters to be scored for the OTU *Coahomasuchus* in the Schoch & Desojo (2016) dataset. Therefore, we conducted a new analysis incorporating recently published anatomical information from other studies (Heckert, Fraser & Schneider, 2017; Schoch & Desojo, 2016; Parker, 2016a), and more character scorings for *C. chathamensis* (Heckert, Fraser & Schneider, 2017). The resulting matrix contains 24 taxa and 44 characters, with the same taxa as Schoch & Desojo (2016), except for the addition of *Gorgetosuchus* (Heckert et al., 2015). We also incorporated all scoring updates from Heckert, Fraser & Schneider (2017) and were able to score *Coahomasuchus* for two of the seven new cranial characters introduced by Schoch & Desojo (2016). We coded *Coahomasuchus* as having a postorbital ventrally extended to form part of ventral orbit margin (state 1) and infratemporal fenestra excluded by postorbital-quadrojugal contact (state 1) for Schoch & Desojo’s (2016) characters 39 and 43 respectively.

**Figure 4:**
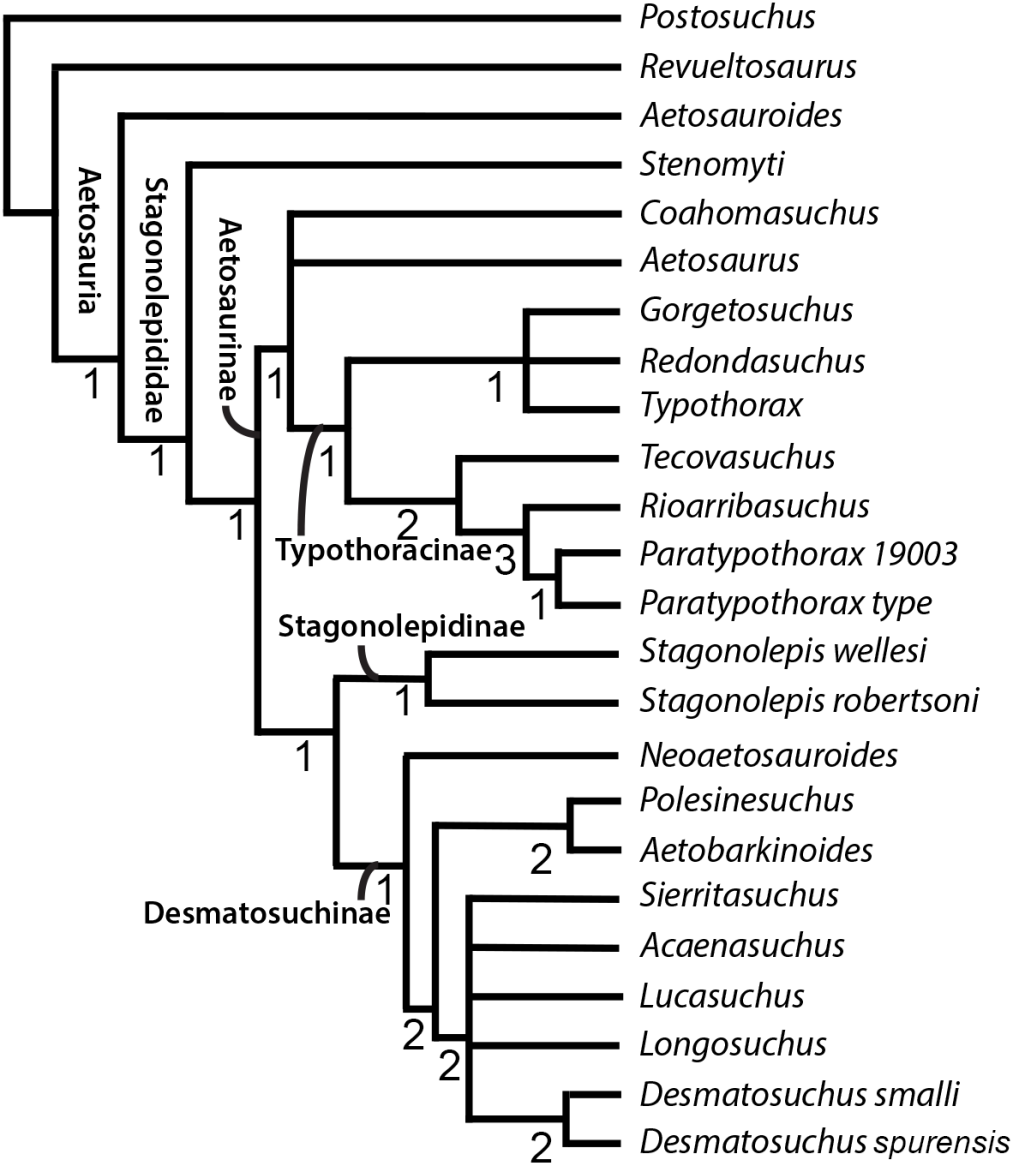
Strict consensus tree of Aetosauria prior to Iterative PCR. Strict consensus of updated matrix (100 steps) from Schoch & Desojo (2016) including *Gorgetosuchus* and updated scorings for other taxa following Heckert et al. (2015). *Coahomasuchus* is recovered in a polytomy with *Aetosaurus* and Typothoracinae, as in Parker (2016a). * Aetobarbakinoides* (and possibly *Polsinesuchus*) may be removed from Desmatosuchinae once more osteoderm characters can be scored. *Aetosaurus* is far removed from a monophyletic * Paratypothorax* in this analysis. Bremer support values are given below nodes.

**Figure 5:**
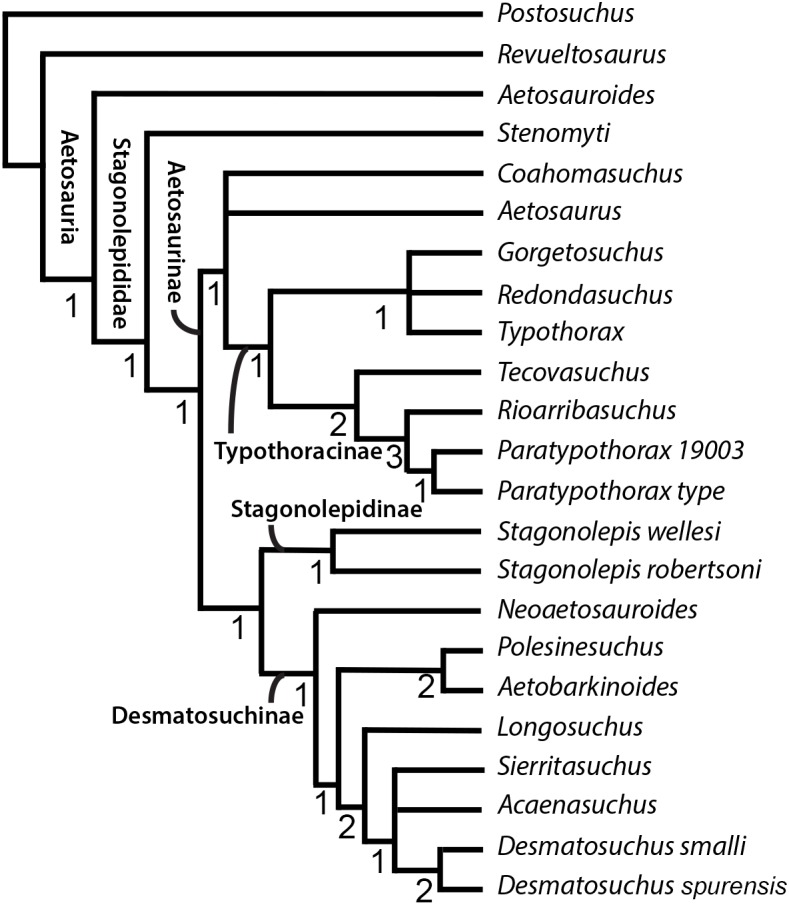
Strict consensus tree of Aetosauria after Iterative PCR. Strict consensus of revised consensus tree from Schoch & Desojo (2016) with *Lucasuchus* pruned through PCRPRUNE command. *Coahomasuchus* remains in a polytomy with *Aetosaurus* and Typothoracinae. Bremer support values are given below nodes.

Cladistic analysis was performed using TNT 1.5 (Goloboff, Farris & Nixon, 2008) following the procedure used by Schoch & Desojo (2016) to ensure as accurate a comparison as possible to see the effects of the updated scorings. Specifically, we performed a traditional search with 50 replications of Wagner trees (with random addition sequence), followed by the TBR branch swapping algorithm (holding 10 trees per replicate) (Schoch & Desojo, 2016). All MPTs were captured in the first round of TBR swapping. Zero-length branches were collapsed following Rule 1 of Coddington & Scharff (1994). To ensure the use of TNT 1.5 rather than TNT 1.1 would not alter results, first we recreated the analysis from Schoch & Desojo (2016), exactly replicating the results of the nine most parsimonious trees (MPTs) of 95 steps and *Coahomasuchus* as the wild card (labile) taxon prior to running the analysis with the updated and corrected matrix. We then ran a more rigorous analysis using 500 replications and holding 20 trees per replicate, yet recovered the same MPTs. We assessed results by generating strict consensus trees prior (Fig. 4) and subsequent to identifying labile taxa via iterative restricted positional congruence (iterative PCR, Pol & Escapa, 2009) and Bremer support values (Bremer, 1994). The strict consensus generated after PCR is reproduced in Fig. 5. Tree figures were generated using Adobe Illustrator.

## Results

After examining the NCSM collections, we were able to refer at least 33 additional fossils to *C. chathamensis* (Table 1; Fig. 3). Importantly, this includes several limb bones (radius and fibula) in addition to several new paramedian and lateral osteoderms. We use the same features as Heckert, Fraser & Schneider (2017) for referring the isolated osteoderms, namely the distinctive (autapomorphic) ornamentation of *C. chathamensis*. The assignment of the limb elements is less certain as Heckert, Fraser & Schneider (2017) do not describe a radius or fibula for the holotype. Instead this assignment is based on the bones presence in otherwise monotypic blocks of *C. chathamensis* osteoderms and the assumption is made the limbs do not come from a different aetosaur taxon that does not have osteoderms represented in the blocks. Additionally, most of these specimens, especially those with sequential numbers, were found associated in various quarry blocks. Thus, although there are many referred specimens, for the most part they represent associated bones gleaned from a much smaller number of blocks of matrix.

**Figure 6:**
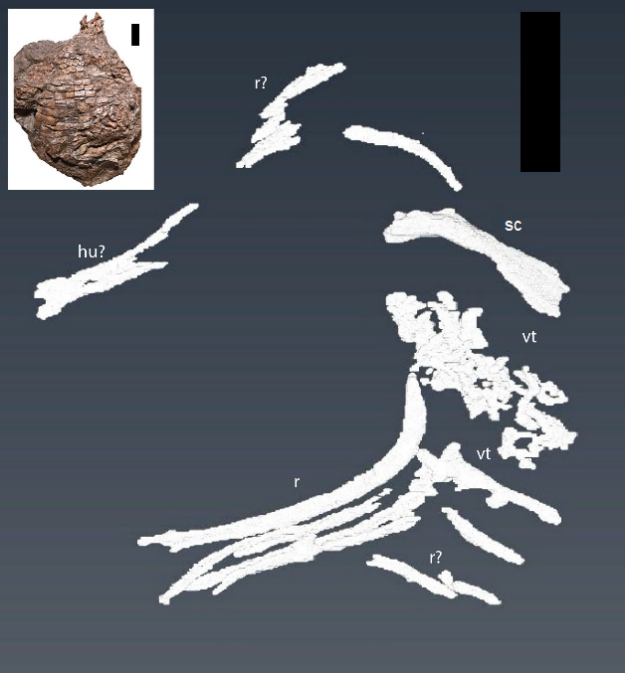
CT reconstruction of internal skeleton with coarser scan (1 mm). Overview (ventral) of segmentation results from coarser scan (1 mm). hu, humerus; r, rib(s); sc, scapula; vt, vertebra(e). Scale bars are 5 cm. Inset shows the holotype specimen in ventral view as in Heckert, Fraser & Schneider (2017, fig. 2).

Two separate CT reconstructions reveal multiple elements not seen on the exterior of the specimen, and provide more information on several other elements that are partially exposed on the surface, but continue into the matrix and disappear from view. Our findings closely match those predicted by Heckert, Fraser & Schneider (2017) in the presence of additional vertebral and forelimb elements. For clarity, we discuss the two different scans separately.

From our initial, low resolution scan we were able to reconstruct a series of articulated vertebrae continuing anterior-medially beneath the vertebrae exposed on the surface, as well as an isolated vertebra (Fig. 6). A limb element, possibly the partially exposed humerus, but more likely the dorsally exposed left scapula due to the strong flexion, is seen on the right anterior portion of the specimen (sc on Fig. 6). The left scapula exposed on the surface was followed and shown to continue down into the matrix (Fig. 6). Additionally, several ribs and other long bone fragments were identified and segmented throughout the specimen (Fig. 6). These are described in greater detail in the following paragraphs.

**Figure 7:**
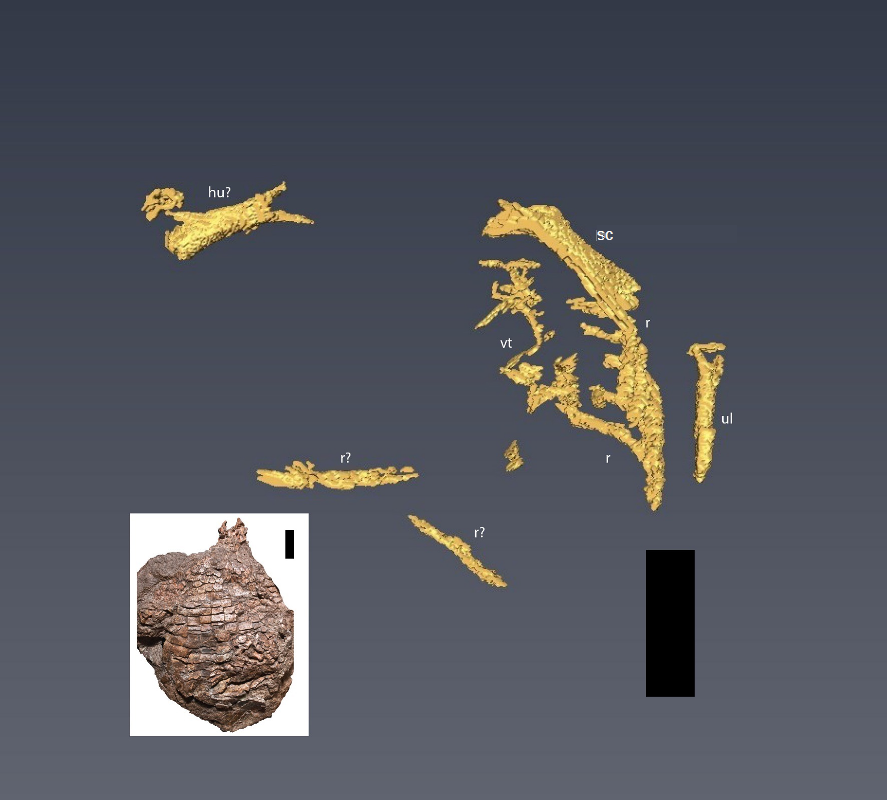
CT reconstruction of internal skeleton with finer scan (0.6 mm). Overview (ventral) of segmentation results from higher resolution scan (0.6 mm). hu, humerus; r, rib(s); sc, scapula; vt, vertebra(e); ul, ulna. Scale bars are 5 cm. Inset shows the holotype specimen in ventral view as in Heckert, Fraser & Schneider (2017, fig. 2).

The second higher-resolution scan captured many of the same elements, including the “limb element”, which appears to be the right humerus, from the original scans with two notable exceptions. The first is a series of apparently articulated ribs along the left margin of the specimen (Fig. 7). The second is a previously unseen element located within the matrix border surrounding the specimen on the left margin, lateral to the ribs, and appears to be the left ulna (Fig. 7). Due to the nature of the beam hardening and priority of previously unseen elements, fewer total elements were reconstructed with this scan.

**Figure 8:**
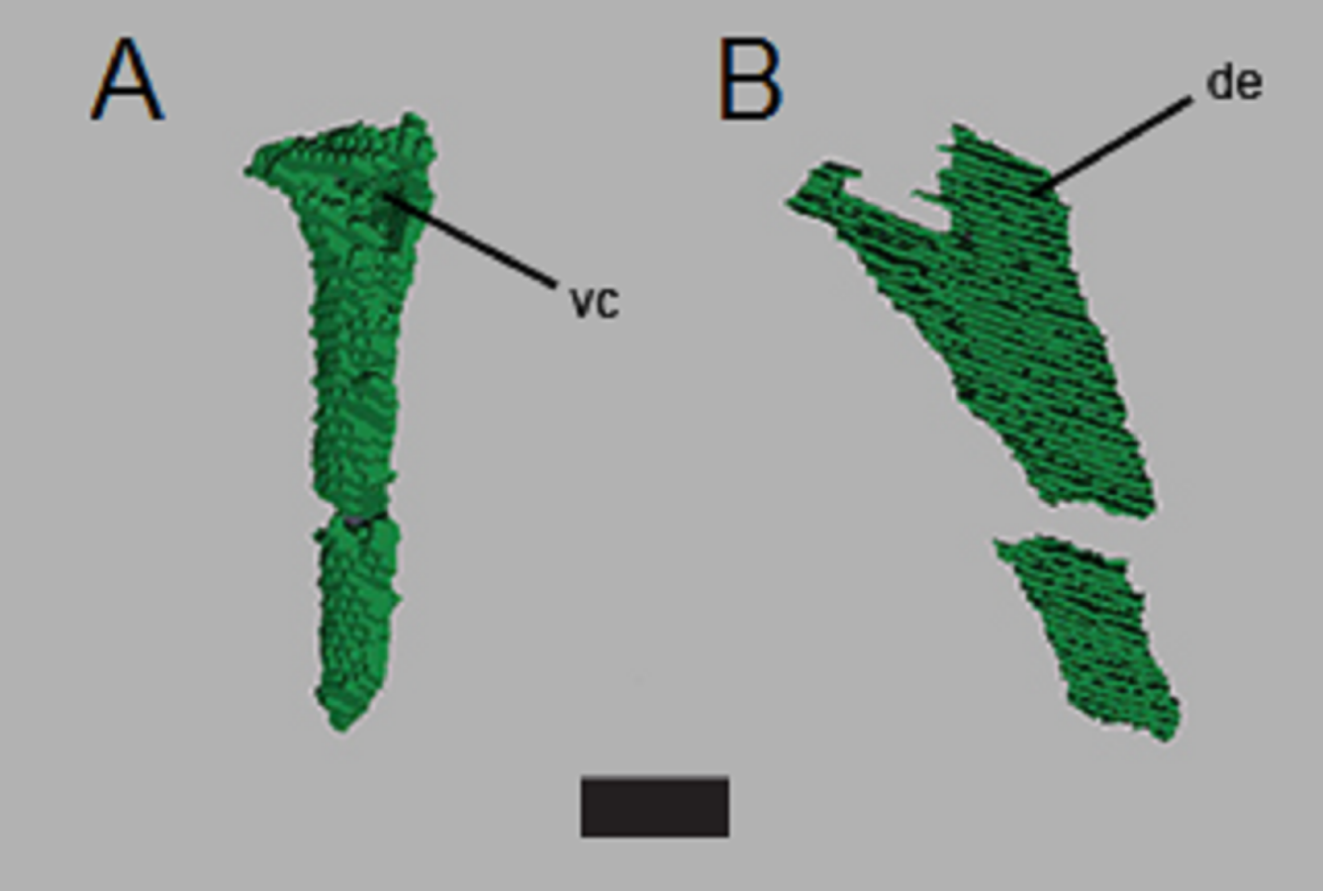
CT segmentation of left ulna. Partial ulna recovered from segmentation data in anterior view (A) and lateral view (B). Proximal end is “up” in the figure. de, dorsal expansion; vc, ventral concavity. Scale bar is 1 cm.

The left scapula is approximately 58 mm long, although the distal end appears to be missing and the preserved length of the right humerus visible on the scans is ∼40 mm (compared to the estimated length of 70 mm for the partially exposed left humerus—Heckert, Fraser & Schneider, 2017). Only part of the distal end of the right humerus is preserved; we were not able to reconstruct detail from the scans. We identify the new limb element from the second scan (Fig. 7) as an ulna due to the position of the bone (lower forelimb), the lack of an ovate, bulbous proximal head found in the radius of *C. kahleorum* (Heckert & Lucas, 1999), and dorsal expansion and ventral concavity (Fig. 8) which can be attributed to the dorsal expansion of the olecranon and the sigmoid notch respectively. These features have been noted in other aetosaurs (e.g., Sawin, 1947; Heckert & Lucas, 1999; Desojo et al., 2013) including *C. kahleorum* (Heckert & Lucas, 1999). Nearly 40 mm of the left ulna is preserved, although there is a fracture approximately two-thirds of the way down the length of the ulna from the visible proximal articular surface, the distal articular surface is no longer present as the reconstruction terminates at the surface. Transversely, the olecranon process is wide (∼10 mm), however, the segmentation on the ventral side does not allow for interpretation of the sigmoid notch beyond noting its presence.

Centra lengths in Fig. 6 range between ∼14 and 16 mm, encapsulating the value (∼15 mm) reported by Heckert, Fraser & Schneider (2017) for the exposed dorsal centra. These lengths are comparable to the parasagittal lengths of nearby osteoderms (∼13–20 mm), and fit with the common 1:1 ratio of vertebrae to osteoderms (Walker, 1961; Long & Murry, 1995; Desojo et al., 2013; Parker, 2016a; Heckert, Fraser & Schneider, 2017). There appear to be at least three, and possibly four, articulated vertebrae not exposed on the surface, as is an additional, isolated, vertebra (Fig. 6). Five articulated ribs are visible in the second scan (Fig. 7), another complete rib is visible from the original scan (Fig. 6), and numerous small fragments can be found in both scans.

The phylogenetic analysis with updated character scorings resulted in 4 MPTs, with a strict consensus tree of 100 steps (Fig. 4). We recovered *Coahomasuchus* in a polytomy with *Aetosaurus* and Typothoracinae, in contrast to Schoch & Desojo’s (2016) result that found *Coahomasuchus* as highly labile and Heckert et al.’s (2015) result of *Coahomasuchus* as a non-stagonolepidid aetosaur. Additionally, iterative PCR identified *Lucasuchus* as a wild card taxon, creating a polytomy within Desmatosuchinae. Excluding *Lucasuchus* and generating an improved strict consensus tree with 2 MPTs and 98 steps, results in slightly improved topology within Desmatosuchinae (Fig. 5). These results are more congruent with a recent study by Parker (2016a) and Parker (2016b) using a larger character set, which also recovered *Coahomasuchus* in a polytomy with Typothoracinae. In each of our four MPTs, however, *Coahomasuchus* is recovered as sister to *Aetosaurus* and Typothoracinae, and is recovered in a hard polytomy within the strict consensus. Surprisingly, *Gorgetosuchus* is recovered within Typothoracinae, a novel result as previous analyses have recovered it as a basal desmatosuchine (Heckert et al., 2015; Parker, 2016a).

## Discussion

To date, no study has attempted to “remove” the osteoderm armor of an aetosaur using CT imaging, nor has this been attempted in other armored fossil groups, although individual osteoderms of dinosaurs (e.g., Curry Rogers et al., 2011) and modern armored animals, such as armadillos and pangolins (e.g., Kawashima et al., 2015) have been imaged with CT technology. This first look under the articulated osteoderms of an aetosaur produced as many challenges as successes. Although several elements could be reconstructed from beneath the osteoderms and within the surrounding matrix, the quality of the reconstructions does not allow for additional character scoring, or even much in the way of qualitative description. Much of the non-osteoderm postcrania that logically should have been present (i.e., additional vertebrae, ribs, other forelimb elements, and the pectoral girdle) were not visible in the scans. Whether this was the result of low resolution in the scans, or the taphonomic removal of elements is unclear. There is evidence of post-mortem alteration of elements in the specimen, including several vertebrae external to the ventral osteoderms, and the movement of right paramedian osteoderms caudally (Heckert, Fraser & Schneider, 2017, fig. 2). The lack of resolution in the scans is due to a combination of compression of the specimen and lack of clear density differentiation between the fossils and the encasing matrix. The dorso-ventral compression of the specimen displaced or damaged several elements, as demonstrated by the multiple thin bone fragments found throughout the carapace (Fig. 6). This also forced the osteoderms into close proximity with the internal skeleton, rendering differentiation of osteoderm and endoskeletal bone difficult. The dense matrix of iron-rich sandstone and conglomerate, as well as the thorough mineralization of the bone, results in a very small density difference between the fossil bones and the surrounding rock. Furthermore, the presence of iron-rich nodules caused beam hardening in both scans.

Despite these complications, several new bones were digitally uncovered (ribs, vertebrae, left ulna, right humerus, left scapula), and others were followed into the matrix (dorsal vertebrae). This result indicates the potential to reveal important morphological data in similarly preserved aetosaurs and other armored animals using CT reconstructions, especially in specimens with greater density differences between the bone and the encasing matrix. A possible candidate is a specimen of *Aetosaurus* from the Sanford Formation (NCSM 11756) described by Lucas, Heckert & Huber (1998). The specimen is an articulated, partial tail (Lucas, Heckert & Huber, 1998, text-fig. 4), which may present a simpler subject; however, the rock density may also render this a difficult subject as well. An additional candidate would be the holotype specimen of *C. kahleorum* (NMMNH P-18496), which could yield phylogenetically informative data for the species and genus, and the specimen may be preserved in a less dense unit of rock than the Sanford sub-basin material (Heckert & Lucas, 1999).

Our phylogenetic analysis most closely matches the results of Parker (2007), Parker (2016a), Parker (2016b) and recovers all five of the recognized major clades of aetosaurs: Stagonolepididae, Aetosaurinae, Stagonolepidinae, Desmatosuchinae (Heckert & Lucas, 1999; Heckert & Lucas, 2000), and Typothoracinae (Parker, 2007). As in Parker (2016a), *Coahomasuchus* is recovered in a polytomy with *Aetosaurus*, as members of Aetosaurinae, and/or as a basal member of Typothoracinae when compared to Schoch & Desojo’s (2016) analysis (Figs. 4 and 5). This appears to be the result of lacking data resolution that would allow the sister taxon relationship with Typothoracinae to be determined. If *Coahomasuchus* is a typothoracine, then it is the stratigraphically oldest one, and provides further evidence of an initial diversification of aetosaurs prior to the early Late Triassic (Nesbitt, 2003; Nesbitt, 2011). Furthermore, with a width:length ratio of homologous dorsal paramedian osteoderms of ≥3.5:1, the Pekin Formation *Coahomasuchus* is one of the stratigraphically oldest occurrences of a wide bodied aetosaur (Heckert, Fraser & Schneider, 2017). Finally, the increased stability of *Coahomasuchus* with the inclusion of updated and revised scorings demonstrates the importance of using the most recent and complete data (Parker, 2016a).

The more nested position of *Aetosaurus* within the aetosaur tree does not fit with early phylogenetic analyses of Aetosauria (Parrish, 1994; Heckert, Hunt & Lucas, 1996; Heckert & Lucas, 1999), yet agrees with more recent work placing it within Stagonolepididae (Parker, 2007; Parker, 2016a). A novel result of our analysis is that it pulls *Stenomyti* outside of Stagonolepididae, unlike other recent analyses (Schoch & Desojo, 2016; Parker, 2016a). The topology of Desmatosuchinae within our analysis resembles that of Schoch & Desojo (2016), with the exception of the polytomy caused by the labile position of *Lucasuchus* (Figs. 4 and 5). We also recover both *Neoaetosauroides* and *Aetobarbakinoides* within Desmatosuchinae rather than early diverging stagonolepids, as in previous studies (Heckert et al., 2015; Schoch & Desojo, 2016; Parker, 2016a). These results do not greatly change accepted aetosaur relationships, however, our results differ from Parker’s (2016a) placement of *Polesinesuchus* within Stagonolepidinae rather than Desmatosuchinae (Fig. 4).

Stagonolepidinae, as recovered in our analysis, differs from the Heckert & Lucas’s (2000) definition of *Coahomasuchus kahleorum* and *Stagonolepis robertsoni*, yet reflects the results of Schoch & Desojo’s (2016) more recent analysis. Contrary to Parker (2016a), we recover *Stagonolepis robertsoni* and *Stagonolepis wellesi* (*Calyptosuchus wellesi*) as sister taxa in a Stagonolepidinae clade, supporting the hypothesis of synonymizing the two in a single genus, *Stagonolepis* (Heckert & Lucas, 2002). This could be the result of limited taxonomic sampling as the matrix we used did not include the Polish *Stagonolepis olenkae* nor the recently described *Scutarx deltatylus* either of which may replace *Stagonolepis robertsoni* as sister to *Stagonolepis wellesi* (*Calyptosuchus wellesi*) (Parker, 2014; Parker, 2016b). However, the placement of *Aetosauroides* as a basal aetosaur within our analysis supports the argument that it should not be considered a junior synonym of *Stagonolepis* (Desojo & Ezcurra, 2011; Parker, 2016a).

The placement of *Gorgetosuchus* within Typothoracinae, in a polytomy with *Redondasuchus* and *Typothorax*, is based on three osteoderm characters (characters 16, 17, 23—see below). This contrasts with other recent phylogenetic analyses that included *Gorgetosuchus* (Heckert et al., 2015; Parker, 2016a) and found it most similar to *Lucasuchus* and *Longosuchus*, although these similarities were also necessarily based entirely on osteoderm characters (Heckert et al., 2015; Parker, 2016a). All three of the synapomorphies (characters 16, 17 and 23 of Parker, 2007, as used by Schoch & Desojo, 2016) that unite *Gorgetosuchus* with *Redondasuchus* and *Typothorax* also characterize some desmatosuchines. Characters 16 and 17 are osteoderm ornamentation characters; *Gorgetosuchus, Redondasuchus* and *Typothorax* exhibit random patterning on paramedian osteoderms (character 16) and small subcircular pits on the paramedian osteoderms (character 17) (Schoch & Desojo, 2016). Character 23 unites all three taxa on the basis of presacral paramedian osteoderms that are strongly flexed ventrally (Schoch & Desojo, 2016). Furthermore, if the dorsal armor of *Gorgetosuchus* were more complete, several more characters of the armor (e.g., characters 25 and 37 of Parker, 2007) could be coded and, based on the preserved cervical specimens, would likely score more similarly to *Longosuchus* and *Desmatosuchus*, assuming that overall osteoderm ornamentation and proportions were to remain consistent further dorsally, but are necessarily coded as “?” because the homologous osteoderms are not preserved. The consistency indices (CI) of these three characters were analyzed in Mesquite and found to be low (character 16 = 0.25, character 17 = 0.33, character 23 = 0.2) suggesting a high degree of homoplasy. However, other characters incorporated in the analysis, including non-osteoderm characters, also have low CI values. Parker (2016a) noted that different character suites (e.g., cranial, osteoderm, postcranial) can give conflicting phylogenetic signals. This becomes a greater issue in datasets attempting to investigate the evolutionary relationships of taxa known exclusively from osteoderms, such as *Gorgetosuchus, Redondasuchus, Sierritasuchus, Acaenasuchus, and Rioarribasuchus*, and taxa with no osteoderm characters scored, such as *Aetobarbakinoides* (Desojo, Ezcurra & Kischlat, 2012). With additional non-osteoderm material of *Gorgetosuchus* this discrepancy should be resolved, and the placement of *Gorgetosuchus* will likely stabilize (Heckert et al., 2015).

## Conclusions

In order to investigate the interrelationships of aetosaurs and to improve our understanding of aetosaur skeletal anatomy we utilized additional fossil material of *C. chathamensis* and CT reconstructions. From the additional material in the NCSM collections, we were able to assign over 30 additional specimens to *C. chathamensis*.

One avenue to improve the accuracy of aetosaur phylogenetic trees suggested by Parker (2016a) is understanding ontogenetic changes in aetosaurs and integrating CT and histologic data into these analyses. As we have demonstrated, CT scanning is a powerful tool and can obtain useful data even in less than ideal circumstances. CT has the ability to reveal informative internal anatomical characters, potentially reveal ontogenetic information (e.g., Cerda & Desojo, 2011; Taborda, Cerda & Desojo, 2013; Scheyer, Desojo & Cerda, 2014), such as the degree of neurocentral suturing (Parker, 2016a), and insights into intraspecific variation (Taborda, Heckert & Desojo, 2015).

Based upon the predictions of Heckert, Fraser & Schneider (2017), we expected the CT reconstructions to reveal additional vertebrae and forelimb elements. Indeed, the segmentations revealed several articulated vertebrae, one isolated vertebra, the left scapula, part of the right humerus, and the left ulna. Heckert, Fraser & Schneider (2017) correctly predicted that either the left ulna or radius was present posterior to the left humerus on the ventral side; our data reveal this to be the ulna. Additionally, we were able to reconstruct several ribs, both thoracic and cervical, including several that were still articulated and some isolated. We found less of the right forelimb than predicted by Heckert, Fraser & Schneider (2017), who speculated that the lower right forelimb may be covered by appendicular osteoderms on the ventral surface.

This study echoes many of the difficulties in aetosaur phylogenetics encountered by previous researchers. The incomplete record of many aetosaur taxa, including a lack of overlapping fossil elements of some taxa and homoplasy in elements that are recovered, results in weakly supported relationships (Harris, Gower & Wilkinson, 2003; Parker, 2007; Parker, Stocker & Irmis, 2008; Desojo, Ezcurra & Kischlat, 2012; Parker, 2016a). The ease in which taxa can shift positions in the phylogeny is represented by the genus *Coahomasuchus*, which has been recovered in four different positions in three recent studies compared to this analysis (Heckert et al., 2015; Schoch & Desojo, 2016; Parker, 2016a). We combined the new data available for *Coahomasuchus* from Heckert, Fraser & Schneider (2017) with the recommended scoring updates of Heckert et al. (2015) to reevaluate the phylogenetic relationships of *Coahomasuchus*. Both replicating Schoch & Desojo (2016) analysis, and running an additional analysis with more rigorous search parameters, resulted in the same, unique topology for Aetosauria, with *Coahomasuchus* forming a polytomy with *Aetosaurus* and Typothoracinae, although in all four MPTs *Coahomasuchus* is sister to *Aetosaurus* + Typothoracinae. Our analysis contained several novel results, notably the placement of *Gorgetosuchus* within Typothoracine and the wild-card taxon status of *Lucasuchus*.

##  Supplemental Information

10.7717/peerj.4368/supp-1 Supplemental Information 1 Phylogenetic character descriptions List of character descriptions used in phylogenetic analysis. Characters 1–37 from Parker (2007) and characters 38–44 added by Schoch & Desojo (2016).

10.7717/peerj.4368/supp-2 Supplemental Information 2 Data matrix used for TNT analysis Complete set of character scorings for taxa used for the phylogenetic analysis in this study. Provided in .txt format for ease of editing and use in multiple programs.

## List of abbreviations

NCSM: North Carolina Museum of Natural Sciences, Raleigh, North Carolina, USA
NCSU: North Carolina State University, Raleigh, North Carolina, USA
NMMNH: New Mexico Museum of Natural History and Science, Albuquerque, New Mexico, USA

## Additional Information and Declarations

### Data Availability

The following information was supplied regarding data availability: The raw data is provided in the Supplemental Files.
