# Supplementary material for: Under the armor: X-ray computed tomographic reconstruction of the internal skeleton of Coahomasuchus chathamensis (Archosauria: Aetosauria) from the Upper Triassic of North Carolina, USA, and a phylogenetic analysis of Aetosauria
Source: PeerJ. 2018 Feb 13;6:e4368. doi: 10.7717/peerj.4368 (PMC5815331; doi:10.7717/peerj.4368)
Supplement: Supplemental Information 1 — List of character descriptions used in phylogenetic analysis. Characters 1–37 from Parker (2007) and characters 38–44 added by Schoch & Desojo (2016). [file peerj-06-4368-s001.docx]

**Appendix – Character list**

1. **Premaxilla morphology.** Anterior end tapering (0); mediolaterally expanded in a shovel-like shape (1). (modified from Parrish, 1994:3; Heckert and Lucas, 1999) Parker (2007: 1).

2. **Dentition.** Teeth mediolaterally compressed and recurved (0), teeth bulbous and conical with recurved tips (1), teeth bulbous and conical with straight tips (2).

3. **Dentary teeth.** Anterior part of the dentary with teeth (0) or edentulous (1).

4. **Premaxilla dentition.** Present, reaching the anterior tip of the alveolar margin (0); present, but the premaxilla is anteriorly edentolous (1); completely absent (2). (modified from Parrish, 1994: 3)

5. **External nares.** Shorter than (0) or longer than (1) antorbital fenestra.

6. **Supratemporal fenestra.** Dorsally exposed (0) or lateral (1).

7. **Jugal.** Not downturned (0) or downturned (1). Ventral margin either straight horizontal (0), or sloping posteroventrally (1), or strongly downturned, aligned at 45° (2).

8. **‘Slipper-shaped’ mandible.** Absent (0) or present (1).

9. **Dentary tooth count.** Nine or more (0) or fewer than nine (1).

10. **Transverse processes of dorsal vertebrae.** Short, less than twice as wide as the centrum (0) or elongate, buttressed ventrally (1).

11. **Presacral neural spine.** High (0) or generally low, less than height of centrum (1).

12. **Cervical centra.** Keeled ventrally (0) or unkeeled (1).

13. **Anterior bars on dorsal and lateral osteoderms.** Absent (0), weakly raised bar (1), strongly raised bar (2).

14. **Width to length ratio of widest dorsal paramedian osteoderms.** Maximum of less than 3.5/1 (0), 3.5/1 or more (1).

15. **Shape of cervical dorsal paramedian osteoderms.** Wider than long (0) or longer than wide (1).

16. **Patterning of paramedian osteoderms.** Radiate (0) or random (1).

17. **Ornamentation of paramedian osteoderms**. Consists of mixture of pits, elongate pits, grooves and ridges (0) or

small subcircular pits only (1).

18. **Dorsal eminence.** Contacts posterior margin of the paramedian osteoderms majority of the time (0) or almost never (1).

19. **Raised dorsal eminence on cervical and anteriormost paramedian osteoderms.** Absent (0), present (1).

20. **Ventral keel or strut.** Never present (0) or present (1) on some or all paramedian osteoderms.

21. **Cervical paramedian osteoderms**. Dorsoventrally thickened with tongue-and-groove articulations: no (0) or yes (1).

22. **Lateral cervical armour**. Lacks (0) or possesses (1) spikes or horns, that may be extremely elongate (2). This character is polymorphic in *Paratypothorax* where half-grown specimens have state 0, adults state 1.

23. **Flexure of presacral paramedian osteoderms.** None or minimal (0), strongly flexed ventrally (1).

24. **Lateral osteoderms.** Minimum angle of flexion between the dorsal and lateral flanges of the lateral osteoderms: obtuse (0), approximately 90° (1), or strongly acute (2).

25. **Middorsal lateral osteoderms.** Symmetry of dorsal and lateral flanges of middorsal lateral osteoderms: symmetrical (0), asymmetrical with dorsal flange longest (1), asymmetrical with lateral flange longest (2). We recoded the states in *Aetosaurus* (0>2), *Neoaetosauroides* (0>2), *Paratypothorax* (2>1).

26. **Narrow region (‘waist’) in the carapace anterior to the sacrum.** Present (0) or absent (1). We recoded the states in *Paratypothorax* (1>0).

27. **Fusion of last presacral vertebra into sacrum.** Does not occur (0) or occurs (1).

28. **Pelvic and anterior caudal lateral osteoderms.** Roughly equant in width and length and possessing a sharp medially situated keel (0), or roughly triangular in lateral view with a semicircular ventrolateral border and a hook-like eminence (1) or rectangular and ventral to a well-developed spine (2).

29. **Dorsal eminence on paramedian osteoderms.** Centralized (0), moderately offset medially (1) or strongly offset medially (2).

30. **Lateral spikes in anterior and mid-dorsal regions.** Not present (0), form a dorsoventrally flattened ‘horn’ (1) or form a conical spine (2).

31. **Number of ventral osteoderm rows**. 10 or more (0), less than 10 (1).

32. **Dorsal eminences on posterior paramedian osteoderms.** In the form of a low pyramid or knob (0) or an elongate spine (1).

33. **Cervical vertebrae.** Extremely shortened anteroposteriorly: no (0) or yes (1).

34. **Posterior margin of paramedian osteoderms.** Strongly bevelled: no (0) or yes (1).

35. **Cervical lateral osteoderms of the sixth row.** Extremely enlarged: no (0) or yes (1).

36. **Dorsal flange of dorsal lateral osteoderms.** Rectangular (0), broadly triangular (1) or tongue-shaped (2).

37. **Mound-like dorsal eminences on anterior dorsal lateral osteoderms.** Absent (0) or present (1)

**Additional characters added by Schoch and Desojo (2016)**

38. **Nasal.** Tapering towards tip (0), or throughout of equal width (1).

39. **Postorbital.** Confined to posterior orbit margin (0), or ventral extended to form part of the ventral orbit margin (1).

40. **Maxilla.** Posterior end sutures broadly with jugal (0), or downcurved and with three separate finger-like processes (1).

41. **Maxilla-Lacrimal.** Suture more or less straight or irregular (0), or with finger-like process of maxilla (1).

42. **Antorbital fossa.** With jugal contribution (0), or formed by the maxilla and lacrimal only, excluding jugal from the margin of the antorbital fenestra (1).

43. **Infratemporal fenestra.** Squamosal enters infratemporal margin (0), or excluded by postorbital-quadratojugal contact (1).

44. **Palpebral bones.** Such extra ossifications in the upper eyelid are absent in the primitive condition (0), and in the derived conditions either a single element is present (1), or three such elements are aligned in a parasagittal row, firmly sutured (2).
